# Supplementary material for: Proteogenomic detection of tumor-specific somatic mutant proteins in urinary extracellular vesicles for non-invasive monitoring of bladder cancer
Source: Front Oncol. 2026 Apr 15;16:1808671. doi: 10.3389/fonc.2026.1808671 (PMC13124499; doi:10.3389/fonc.2026.1808671)
Supplement: Supplementary file 1 [file DataSheet1.docx]

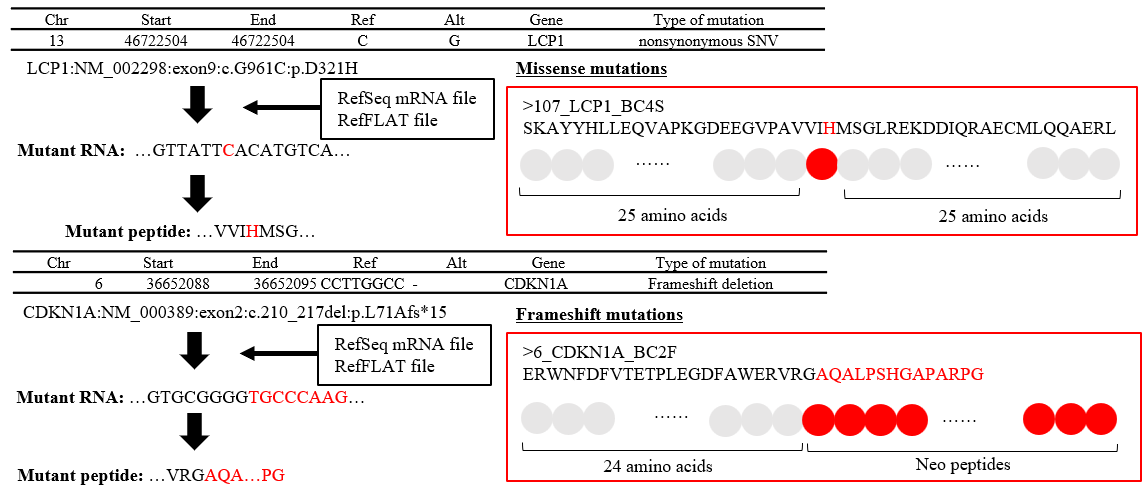


Fig. S1. **Construction of amino acid sequence database for mutant peptides by Neoantimon software.** This software generates mutant peptides by constructing the corresponding RNA sequences using the Refseq mRNA file and refFLAT file. Here, mutant peptide sequences including missense and frameshift mutations were obtained.


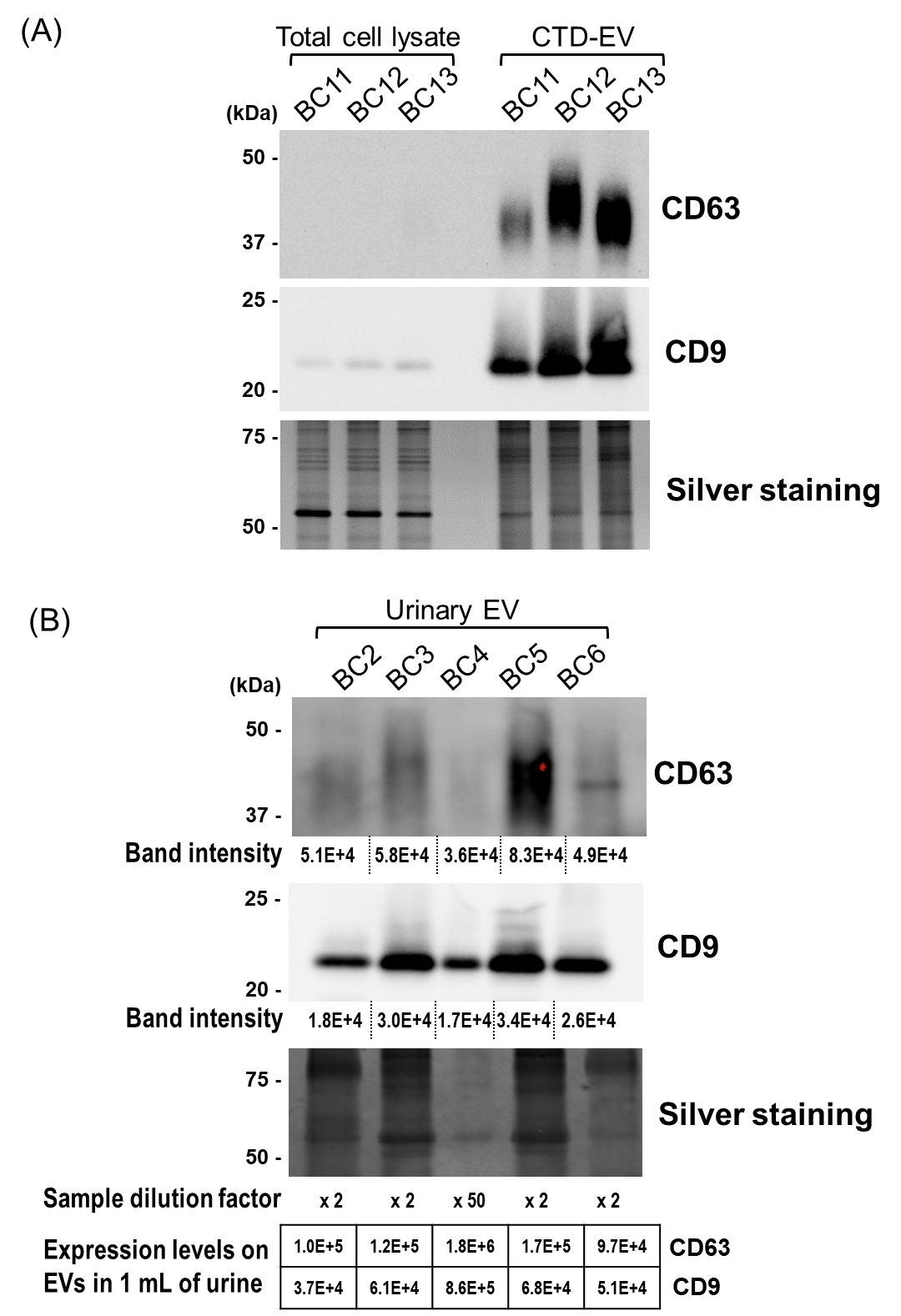


Fig. S2. Evaluation of EV marker proteins by western blotting (A) using before and after EV isolation using cultured tissue exudative extracellular vesicle (CTD-EV). 10 μg of proteins were loaded in each lane which were visualized with silver staining. (B) Western blot analysis and silver staining of urinary EVs isolated using MagCapture beads. Urinary EVs were purified from 1 mL of urine and was loaded per lane (equivalent to 0.5 mL of original urine for BC2, BC3, BC5, and BC6, and 0.02 mL for BC4). Band intensity was measured by Image J software and used to calculate relative expression levels of EV-CD63 or EV-CD9 in 1 mL of urine shown in the bottom table.


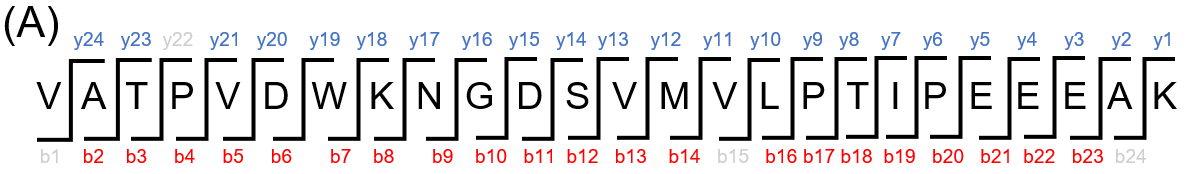


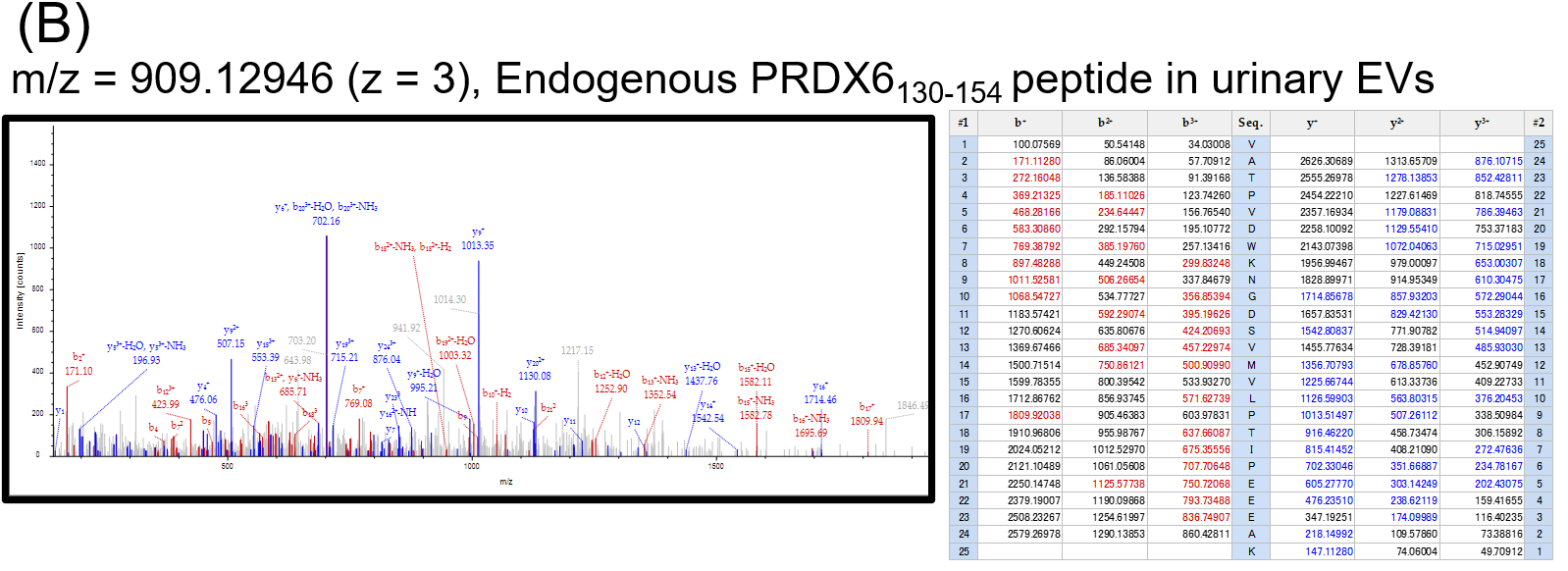


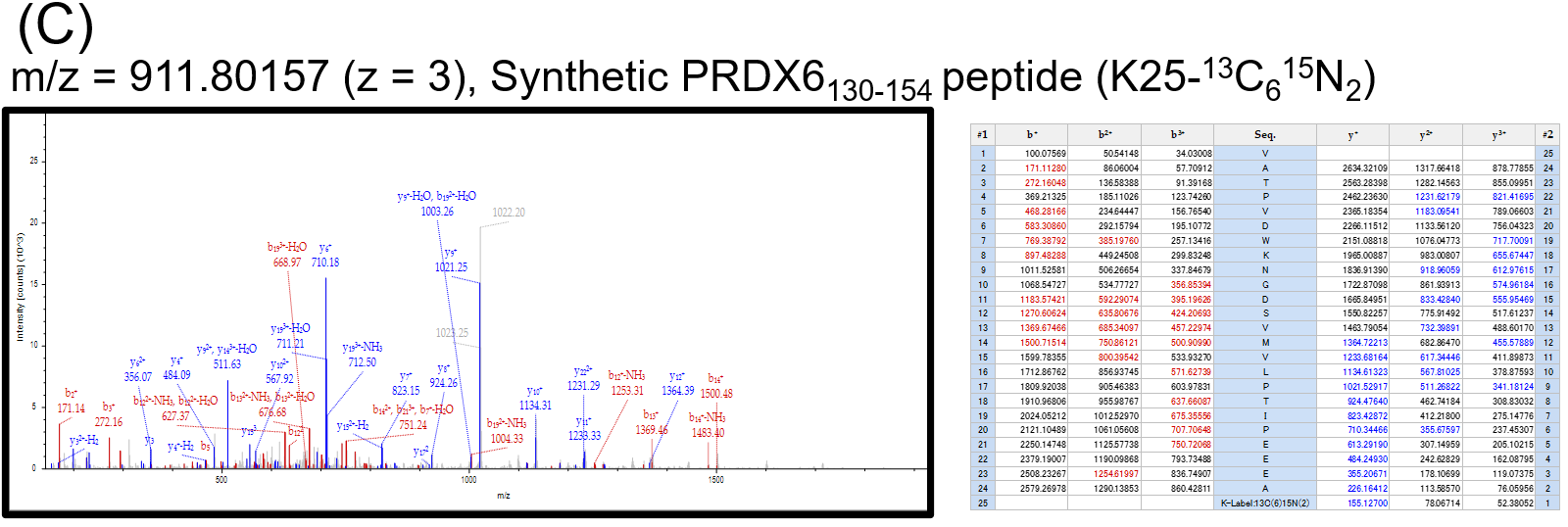


Fig. S3. Technical validation of the mass spectrometric identification of PRDX6_p.D138N peptide. (A) The amino acid sequence of D138N_130-154_ peptide is shown, which was detected from the urinary EV sample of the patient BC2. The b- and y-series fragment ions identified in the Sequest database search were displayed in red and blue letters, respectively. The centroid MS/MS spectrum of endogenous PRDX6_130-154_ peptide detected from urinary EVs (B) or synthetic PRDX6_130-154_ peptide (C) was shown in the left panel. The right matrix demonstrates the sequence coverage in the Sequest database search. The b- and y-series fragment ions identified in the Sequest database search were displayed in red and blue letters, respectively.


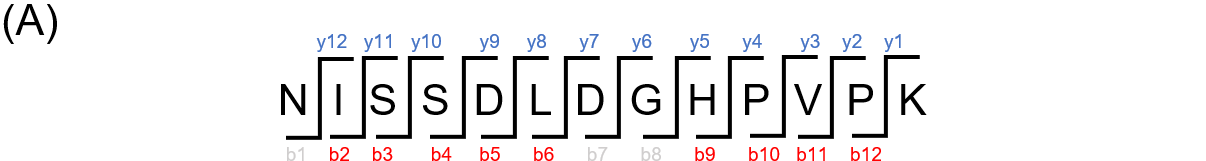


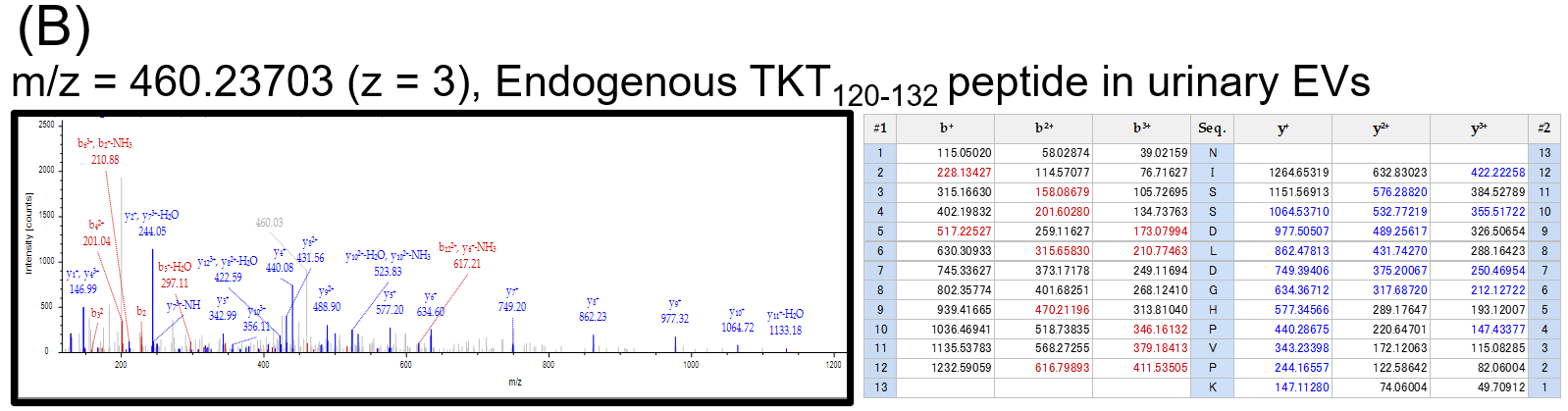


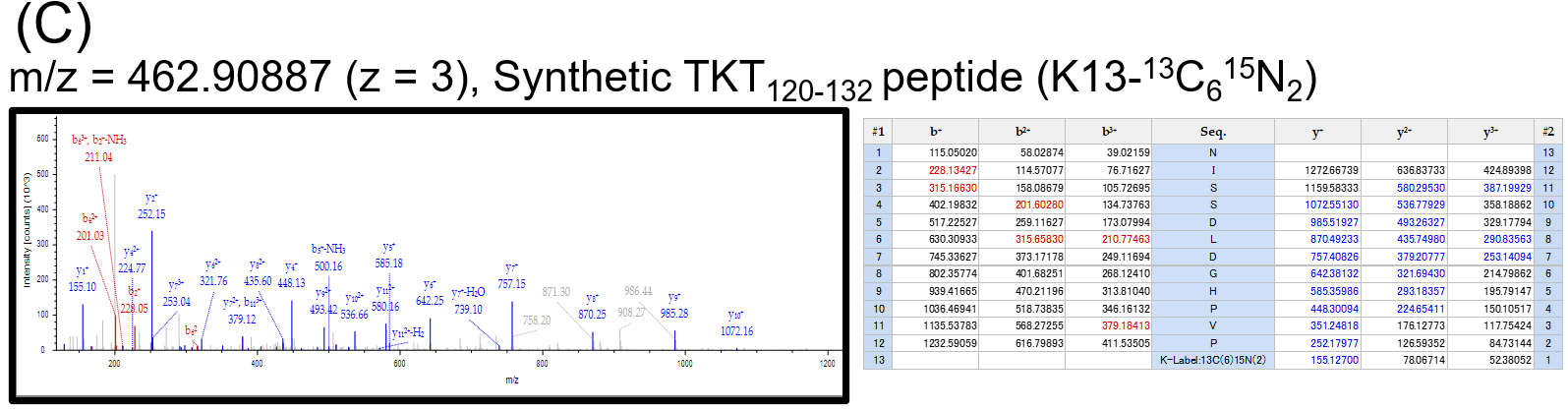
Fig. S4. Technical validation of the mass spectrometric identification of TKT_p.K120N peptide. (A) The amino acid sequence of K120N_120-132_ peptide is shown, which was detected from the urinary EV sample of the patient BC4. The b- and y-series fragment ions identified in the Sequest database search were displayed in red and blue letters, respectively. The centroid MS/MS spectrum of endogenous TKT_120-132_ peptide detected from urinary EVs (B) or synthetic TKT_120-132_ peptide (C) was shown in the left panel. The right matrix demonstrates the sequence coverage in the Sequest database search. The b- and y-series fragment ions identified in the Sequest database search were displayed in red and blue letters, respectively.


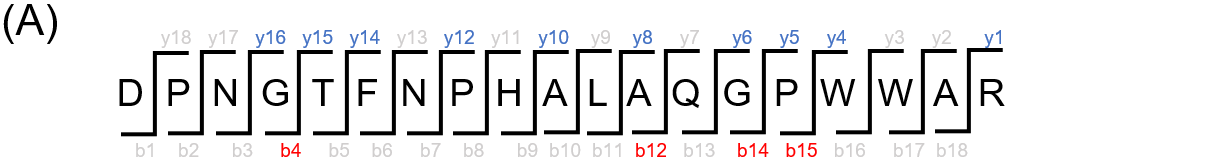

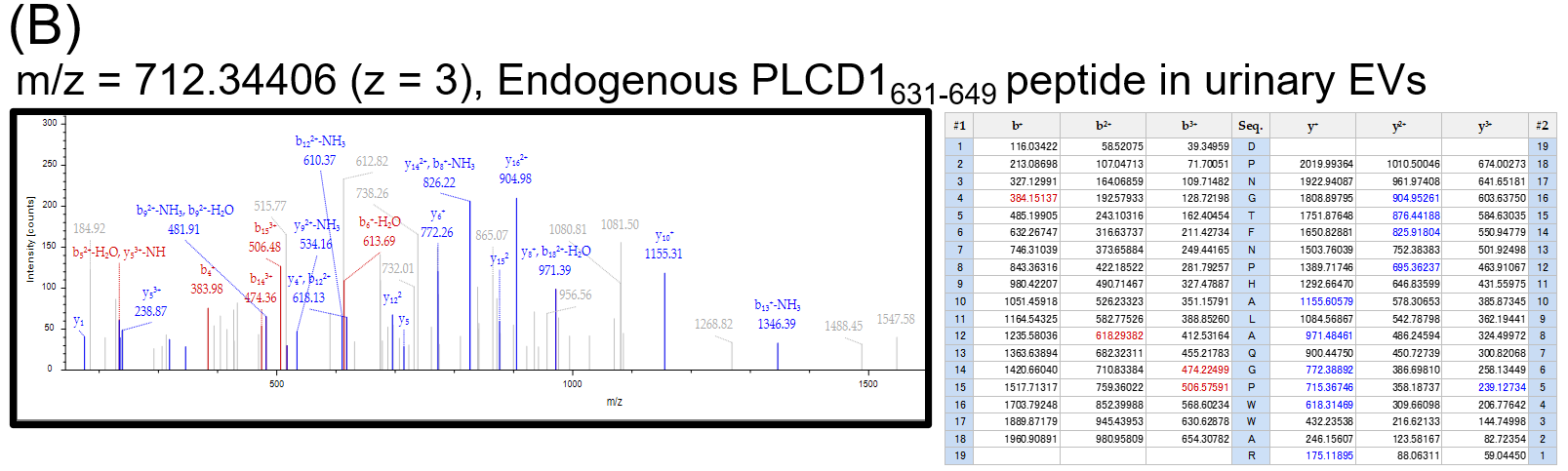

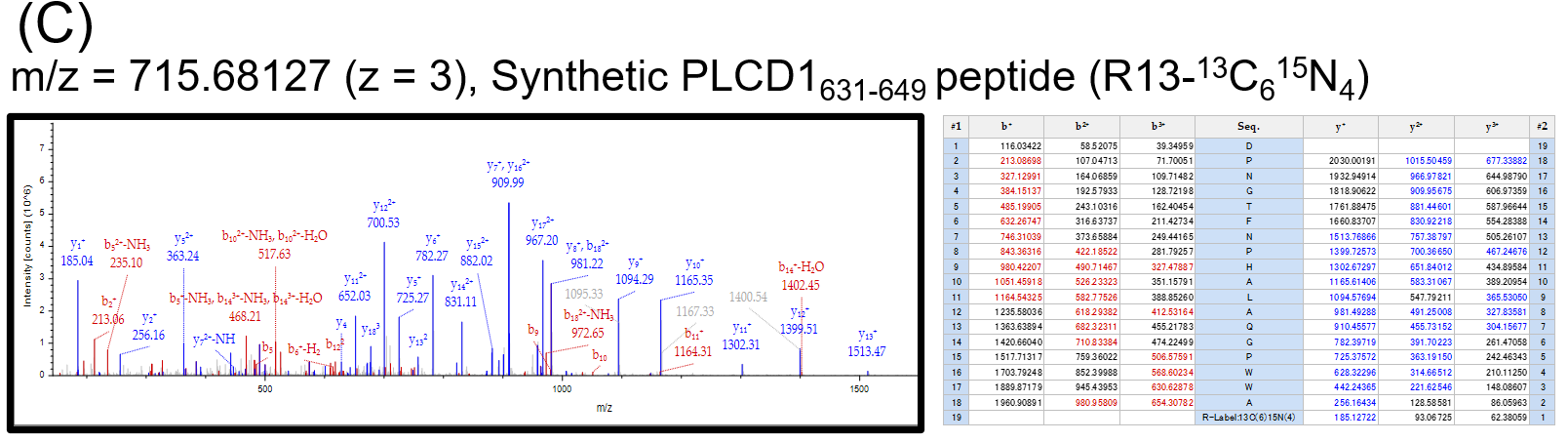
Fig. S5. Technical validation of the mass spectrometric identification of PLCD1_p.R639H peptide. (A) The amino acid sequence of R639H_631-649_ peptide is shown, which was detected from the urinary EV sample of the patient BC4. The b- and y-series fragment ions identified in the Sequest database search were displayed in red and blue letters, respectively. The centroid MS/MS spectrum of endogenous PLCD1_631-649_ peptide detected from urinary EVs (B) or synthetic PLCD1_631-649_ peptide (C) was shown in the left panel. The right matrix demonstrates the sequence coverage in the Sequest database search. The b- and y-series fragment ions identified in the Sequest database search were displayed in red and blue letters, respectively.


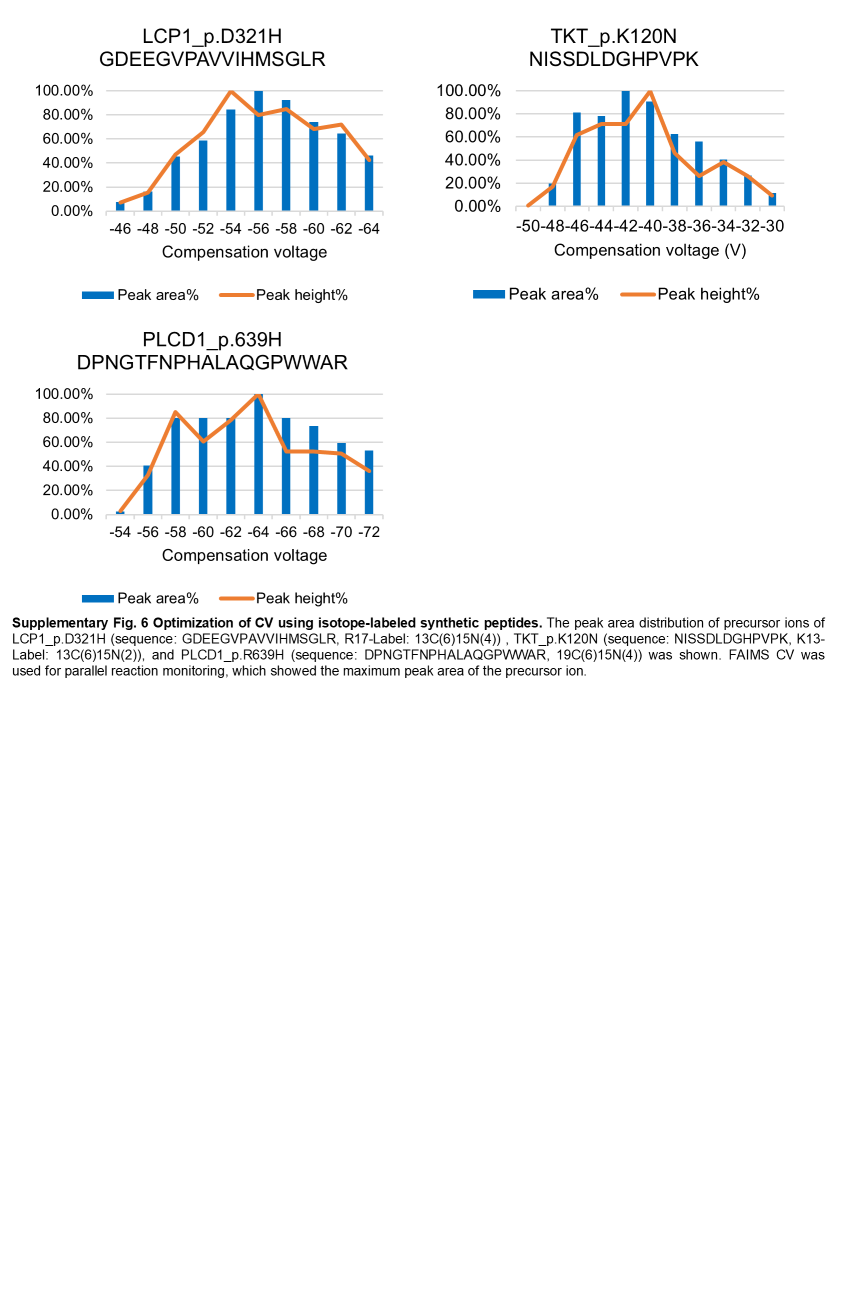


Fig. S6. Optimization of CV using isotope-labeled synthetic peptides. The peak area distribution of precursor ions of LCP1_p.D321H (sequence: GDEEGVPAVVIHMSGLR, R17-Label: 13C(6)15N(4)) , TKT_p.K120N (sequence: NISSDLDGHPVPK, K13-Label: 13C(6)15N(2)), and PLCD1_p.R639H (sequence: DPNGTFNPHALAQGPWWAR, 19C(6)15N(4)) was shown. FAIMS CV was used for parallel reaction monitoring, which showed the maximum peak area of the precursor ion.

**
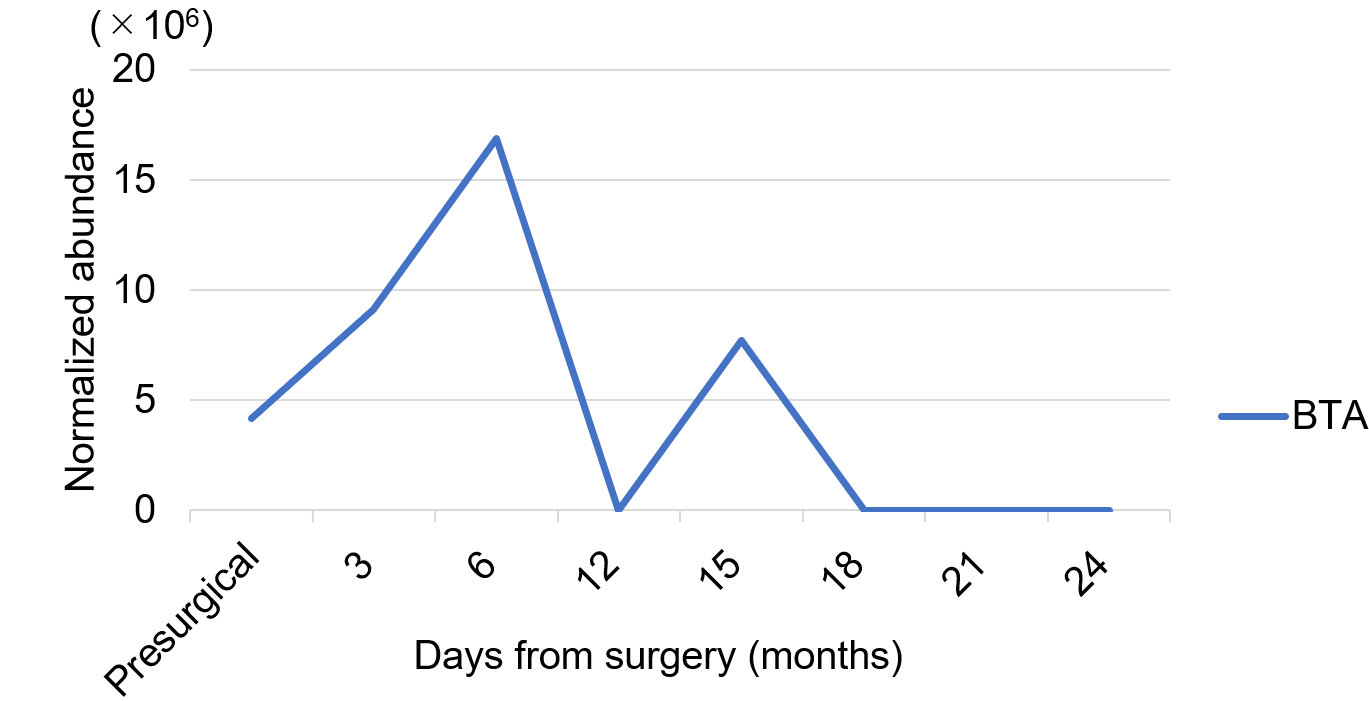
**

**Fig. S7. Longitudinal monitoring of mutant proteins in urinary EVs after surgery.**Normalized abundance of complement factor H, the principal target of the BTA assay, in urinary EVs was measured before surgery and during postoperative follow-up (3–24 months).
